# Supplementary material for: Are carbon emissions trading and green financial instruments synergistic? -Comprehensive quantitative research based on content analysis
Source: PLoS One. 2024 Mar 7;19(3):e0298601. doi: 10.1371/journal.pone.0298601 (PMC10919601; doi:10.1371/journal.pone.0298601)
Supplement: S1 Data — (ZIP) [file pone.0298601.s001.zip › Data/地级市Z_F工作报告.zip!/╕╩╦α/┬ñ─╧╩╨/└┤╫╘╣1⁄2╓┌║┼í╛┬φ┐╦╩2╛▌═°í┐_2019.docx]

各位代表：

  现在，我代表市人民政府，向大会报告政府工作，请予审议，并请各位政协委员和列席人员提出宝贵意见。

2018年工作回顾

  刚刚过去的一年，是我们在习近平新时代中国特色社会主义思想的指引下，开拓进取，奋勇前进，开启新征程的一年；是在经济下行压力加大、脱贫攻坚任务艰巨、自然灾害频发的严峻挑战下，拼搏实干，砥砺前行，开创新业绩的一年；也是在改革开放40周年伟大成就的鼓舞下，牢记使命，继往开来，开辟新局面的一年。一年来，在省委省政府和市委的正确领导下，我们深入学习贯彻习近平新时代中国特色社会主义思想和党的十九大精神，全面落实习近平总书记视察甘肃重要讲话和“八个着力”重要指示精神，牢固树立新发展理念，按照高质量发展要求，深化实施“433”发展战略，统筹推进稳增长、促改革、调结构、惠民生、防风险各项工作，脱贫攻坚成效明显、经济运行稳中有进、社会大局和谐稳定、人民生活持续改善，朝着建成幸福美丽新陇南的目标迈出了新的步伐。

  ——经济运行保持了好势头。在经济下行压力加大的形势下，负重前行、迎难而上，全力推进高质量发展，全市实现生产总值379.2亿元、增长5.8%，第一产业实现增加值71亿元、增长3.7%，第二产业实现增加值74.6亿元、增长4.7%，第三产业实现增加值233.6亿元、增长6.8%，我们在全省高质量发展排名居于第六位，保持了稳中有进的良好势头。

  ——脱贫攻坚取得了好成绩。在任务十分艰巨的情况下，全市上下万众一心，攻坚克难，着力解决“两不愁三保障”问题，主要指标全面提升，全年完成脱贫13.27万人，有713个贫困村脱贫退出，贫困发生率下降到7.8%。两当县顺利实现脱贫摘帽，成县、徽县达到整县脱贫退出标准。合作社控股富民公司带贫的“宕昌模式”得到省政府充分肯定并在全省推广，我市荣获2018年全国“十佳精准扶贫创新城市”称号，取得了重大阶段性成果。

  ——交通实现了立体发展。成县机场正式通航运营，旅客吞吐量累计达到18万人次。兰渝铁路开通动车组，陇南到兰州时间缩短至3小时以内。渭武、两徽高速部分区间基本具备通车条件，武九高速全线开工，全市综合立体交通网络基本形成，出入陇南更多样、更便利、更快捷，“千里之外、朝发夕至”，使陇南发展迈上了快速通道。

  ——城乡建设显出了新魅力。宜居城市、特色小镇、美丽乡村深入推进，城乡治理水平明显提高，新城区建设取得了新进展，老城区改造焕发了新气象，乡村振兴展现出新面貌，城乡变得更干净、更美丽、更宜居了，越来越成为安居乐业的美丽家园，成为旅游康养的理想之地。

  ——大数据发挥了大作用。在全省率先建成了陇南市大数据云计算中心，完善提升了灾情预警、精准扶贫、政务、乡村等多个大数据系统，整合了各行各业，联通了千家万户，在社会治理、防灾减灾、政务服务等方面发挥了重大作用，中央改革办《改革情况交流》专门编发了陇南的做法，省委书记林铎批示在全省推广陇南经验。

  ——农村电商作出了新贡献。坚持把电子商务与特色产业开发深度融合，电商网店遍布百乡千村，特色产品远销东南西北，成为农民群众脱贫致富的好帮手，为陇南经济跨越发展创出了新路子，我市电商扶贫被评为全国产业扶贫十大机制创新典型案例，荣获2018中国智慧三农大会特别大奖。

  ——改革开放迈出了新步伐。全面深化“放管服”改革，大力推行“互联网+政务服务”，优化了营商环境，方便了群众办事。全面推行农村“三变”改革，有效盘活了生产要素，使农村活力变强、农民收入变好。积极扩大对外开放，广泛开展招商引资，成功举办了花椒、苹果、油橄榄、中医药等节会，形成了开放发展的良好局面。

  ——防灾减灾取得了好成效。面对年年有灾、年年受灾的市情，积极预防，奋力抢险，有效应对了暴洪泥石流等重大自然灾害，最大程度减少了灾害损失，有力保护了人民群众生命财产安全，得到了省委省政府的充分肯定。

  ——人民群众得到了更多实惠。切实保障和改善人民群众生活，省市20件民生实事全部办结，城乡公益性岗位使4.3万人受益，城乡居民人均可支配收入分别达到23782元、7012元，增长7.2%、9.8%。城乡教学条件不断改善，医疗医保水平不断提高，兜底保障更加有力，民族宗教工作创新发展，生态环境、安全生产和社会治安持续好转，人民群众的获得感、幸福感、安全感进一步提升。

  回顾过去的一年，经历了艰难困苦。我们能在经济下行压力加大、项目投资增长乏力、风险隐患显现的形势下，保持经济平稳较快增长，一些主要指标进入全省前列十分不易；我们能在脱贫攻坚和抢险救灾任务艰巨繁重的情况下，统筹推进改革发展稳定各项工作，补短板、强弱项、提质量取得重大进展更为难得。实践再次证明，只要我们上下同心、勇于担当、真抓实干，坚持深化实施“433”发展战略，就一定能够战胜发展中的一切困难和挑战，就一定能够建成幸福美丽新陇南！

  一年来，我们主要做了以下工作：

  我们坚持精准方略，拼命实干补短板，脱贫攻坚成绩显著。始终把脱贫攻坚作为首要的政治任务和最大的民生工程，紧盯目标任务，精准施策发力，拼命攻坚克难，提升短板弱项，顺利完成了阶段性任务。全面提升贫困乡村硬件基础，建设自然村道路468公里，完成农村公路“畅返不畅”整治309公里，实施易地扶贫搬迁1.14万户4.76万人，改造农村危房2万户，解决了142个贫困村2万户贫困户的饮水问题。大力推进产业扶贫，深入落实“一户一策”“一村一品”产业发展计划，积极发展牛羊菜果薯药和区域性特色产业，有效增加了贫困群众收入。培育壮大经营主体，市县组建了产业富民公司，推行“富民公司+合作社+贫困户”的经营模式，组建扶贫车间174家，实现了每个贫困村2个以上合作社全覆盖，北京德青源金鸡产业项目在宕昌县、礼县、西和县落户，涌现出了宕昌县合作社控股富民公司带贫新模式。着力解决合作社人才短缺的问题，出台了三个政策文件，鼓励事业单位人员、未就业大中专毕业生和企业经营管理人才到贫困村领办创办合作社，提升合作社带动能力。在全省率先成立“金控陇南担保公司”，发放特色产业发展工程贷款10.5亿元，落实到户产业扶持资金15亿元。建成贫困村果蔬保鲜库160座，全面消除了集体经济“空壳村”。异地实施新一轮光伏扶贫工程1039户，创建乡村旅游扶贫示范村21个。大力推进电商扶贫，电子商务进农村示范县项目实现全覆盖，创建电商扶贫示范网店1033家、带动贫困人口18万人。积极推进就业扶贫，组织输转贫困劳动力6万多人，落实农村公益性岗位3.85万人，生态护林员9797名。全面落实健康扶贫政策，全市303家定点医疗机构实现了“先看病后付费”和“一站式”结算全覆盖，2000元的报销起付线和85%的报销比例全部实现，全省健康扶贫“五帮两核”现场会在我市召开。扎实推进教育扶贫，有需求行政村幼儿园基本实现全覆盖，义务教育适龄儿童全部在校就读。不断深化东西扶贫协作，在资金投入、人才培养、产业合作、劳务协作等方面加大了力度，青岛市区两级落实帮扶资金3.66亿元，引进项目22个，中央定点帮扶单位落实帮扶资金4350万元。全省“千企帮千村”工作推进会在我市召开，99家企业结对帮扶贫困村合作社，社会帮扶成效明显。

  我们坚持高质量发展，发挥优势调结构，产业效益不断提升。认真贯彻落实省委省政府推动绿色发展崛起的决定，以发展十大生态产业为重点，充分发挥特色优势，积极调整产业结构，高质量发展迈出新步伐。加快发展现代农业，实施冬小麦绿色高效项目17万亩，粮食总产量增长3.48%，粮食安全得到保障。新发展经济林果35万亩，特色产业面积稳定在1000万亩左右，高效露地蔬菜基地、海升集团蓝莓示范园和100个特色产业示范点建设进展顺利，编制完成了60个田园综合体规划，祥宇橄榄油荣获以色列“2018年特拉橄榄油大赛”金奖。新建标准化规模养殖场30个，发展生态放养鸡557万只、中蜂养殖34万箱，我市被评为国家蜂业提质工程示范市。发展壮大中医中药产业，建成中药材标准化基地46万亩，成功举办了中国（甘肃）中医药博览会陇南分会、签约资金14亿元。加快推进工业转型升级，实施市列重点工业项目29个，完成投资31.9亿元，金徽酒业技改、春天药业中药饮片加工等项目建成投产，厂坝公司300万吨扩能、鑫晟源大黄全产业链等12个项目建设进展顺利，新增中小微企业488户，主要工业产品大幅增长，规上工业增加值增长7.1%，实现利润21亿元。（下转第二版) (上接第一版)加快发展全域旅游业，官鹅沟5A级景区创建步伐加快，哈达铺、文县天池、金徽矿业创建为4A级景区。实施武都古今里、金徽酒业景区二期等旅游项目85个，完成投资38.8亿元。康县依托美丽乡村发展全域旅游的经验得到中央肯定。成功举办了乞巧女儿节、白马人民俗文化旅游节、官鹅沟冰雪旅游节，全市旅游接待人数1760万人次，实现旅游综合收入92.4亿元。积极发展通道物流产业，武都吉石坝现代物流园、成县顺通电商物流园二期、宕昌中药材现代仓储物流园等项目顺利实施，全市物流企业达到296家，铁路、公路货运周转量分别增长130%、2.6%，社会消费品零售总额增长7.4%。积极推进大数据建设，建成陇南市情和特色产业遥感分析系统，“陇南乡村大数据”覆盖70%以上家庭。大力发展农村电子商务，网店达到1.4万家，青岛陇南电商体验中心正式运营，陇南农业众筹扶贫模式在全国推广。加快陇南经济开发区建设，审批入园企业30户，完成工业总产值23亿元。加大尾矿资源综合回收利用，金徽矿业、成县祁连山水泥公司被工信部评为全国“绿色工厂”，全省绿色矿山建设现场会在我市召开。

  我们坚持扩大投资，凝聚全力抓项目，发展基础更加坚实。针对投资大幅下降的问题，严格落实投资项目清单管理制和领导干部包抓项目责任制，坚持定期召开项目建设调度会，集中力量推进项目建设，基础设施建设不断完善。实施亿元以上重大项目187个，完成投资120.7亿元，7个省列重大项目完成投资74.1亿元。加快交通建设，渭武、两徽高速进展顺利，武九高速全线开工，建设省道和旅游公路584公里，建成农村公路1416公里。签约PPP项目4个，总投资18.9亿元，全力推进洛门至礼县、文县余家湾至凡昌、康县望关至宁强高速公路等项目前期工作。实施水利建设项目77项，完成投资11.9亿元。完成电信普遍服务试点项目，4G网络覆盖率达到97%。加大城乡建设力度，实施市政基础设施项目88项，完成投资9.35亿元，武都区滨江步道景观改造、东江污水处理厂异地新建、文县文兴廊桥工程、徽县城区集中供热等项目建成投用，东江新区外环路、成县城区污水处理厂升级改造、西和县城区集中供热等项目加快推进。实施棚户区改造项目24个4231套，完成建筑业增加值32.4亿元。加快实施乡村振兴战略，扎实开展全域无垃圾专项治理行动，新建生态文明新农村381个，创建省级美丽乡村示范村21个，许多乡村成为生态宜居的美丽家园。

  我们坚持生态优先，呵护绿色严治理，生态环境持续改善。牢固树立绿色发展理念，持续加强生态文明建设，天蓝地绿的生态环境成为陇南的一大名片。扎实开展“绿盾2018”专项行动，生态红线划定工作全面完成，自然保护区调整有序推进，各类保护地矿业权退出取得阶段性成效，大熊猫国家公园建设进展顺利。大气、水、土壤污染防治取得明显成效，水生态文明城市建设试点任务圆满完成，两当县获得全国生态文明建设示范县称号。深入开展燃煤锅炉、施工扬尘等专项整治，扎实推进中央和省级环保督查反馈问题整改，环境空气质量、地表水监测达标率均居全省第二，单位生产总值能耗、污染减排控制在省定目标以内。大力实施国土绿化行动，植树造林52万亩，退耕还林还草40万亩，打造了一批绿化带、产业带、景观带，森林面积新增1.6万亩，森林覆盖率达到41.95%。全面落实河长制、湖长制，统筹推进河湖环境综合治理和沿河经济发展，康县沿河治理成效明显。深入推进“双查双整”集中行动，尾矿库综合治理有效，全省矿山安全生产工作现场会在我市召开。

  我们坚持内改外联，深化改革促开放，发展活力不断增强。持续深化重点领域和关键环节改革，全方位扩大对外开放，经济社会发展的活力不断增强。深入推进供给侧结构性改革，关闭退出3户煤矿企业，房地产去库存周期缩短为9个月，企业生产经营成本明显下降。深化投融资体制改革，全市金融机构贷款余额658亿元、增长7.2%，金鑫新材料公司在“新三板”挂牌上市，实现我市“新三板”挂牌企业零突破。全面推进“四办”改革，启动“减证便民”专项行动，取消调整下放行政审批事项165项，公布“最多跑一次”事项318项，704项政务服务事项全部可在线办理；统筹推进“多证合一”改革，发放电子营业执照7251户，市场主体达到13.6万户、增长38.2%。全面推进农村“三变”改革，参改农户11.9万户，农村土地确权颁证率97.8%，流转土地123万亩。深化医药卫生体制改革，公立医院全部取消药品加成，分级诊疗制度全面推行，全省基层卫生改革现场推进会在我市召开。加强对外交流合作，深化与白俄罗斯格罗德诺市经贸人文交流，组织参加哈萨克斯坦、阿联酋等国外商品展销活动，外贸出口额增长66.4%。积极举办各类节会，市县主要领导带队赴北京、广州、深圳、青岛等省市广泛开展招商引资活动，签约项目65个、签约资金98亿元，到位资金73.6亿元，越来越多的外地企业在陇南投资创业。

  我们坚持人民至上，倾注真情惠民生，人民生活质量不断提高。始终把排民忧、解民难作为工作的出发点和落脚点，持续加大社会事业和民生保障力度，人民群众得到了更多的实惠。全面落实各项就业政策，城镇新增就业2.07万人，城镇登记失业率控制在4%以内。大力发展教育事业，全面改薄、教师周转房、温暖工程、幼儿园建设等项目建成投用，新建6所城区中小学校，文县、西和县顺利通过国家义务教育发展基本均衡县评估认定，全市义务教育巩固率达到95.1%，城区学校“入学难”“大班额”问题得到有效缓解，普通高考一本、二本上线率分别达到15.2%、32.94%。大力推进健康陇南建设，全面实施医联体建设，居民医保人均财政补助标准提高到490元，建成城乡养老机构8所、老年人日间照料中心120个、医养结合示范点10个，城乡居民养老保险续保率97%，城乡低保和特困供养标准分别提高7.6%、6.3%和7.6%，为农村老年人和留守儿童购买人身意外伤害险656万元，兜底保障水平全面提升。加强科技创新，新认定国家级知识产权优势企业1个、省级高新技术企业3个，建立专家院士工作站、实验室7个。积极推进文化体育事业，建成社区综合文化服务中心22个，新建“历史再现”工程博物馆12个，《阴平古道》纪录片在央视播出；体育基础设施进一步完善，城乡全民健身活动广泛开展，陇南健儿在省第十四届运动会上取得历史最好成绩。全面加强安全生产，深入开展道路交通、建筑施工、食品药品安全专项整治，安全生产形势持续稳定向好。扎实开展“扫黑除恶”专项斗争，积极有效推进信访工作，保持了社会大局安定祥和。

  我们坚持从严管理，转变作风提效能，政府自身建设不断加强。牢固树立“四个意识”，坚决做到“两个维护”，深入推动中央、省委省政府和市委各项决策部署落地见效。严格执行中央八项规定及实施细则精神、国务院“约法三章”、省委省政府和市委各项规定，“三公”经费下降26.5%。扎实开展“转变工作作风改善发展环境建设年”活动，制定出台市政府“十个不准、八个严查”制度，持续整治“四风”，全市营商环境明显好转。自觉接受市人大的法律监督、市政协的民主监督，办理人大代表建议93件、政协委员提案149件。坚持科学民主决策，聘请市政府经济顾问、人才顾问建言献策。成立了廉政监督委员会，加强法治政府建设和党风廉政建设，有效推进了依法行政、清廉行政。同时，我们统筹兼顾，真抓实干，全面推进其他领域的各项工作，不断取得了新的成绩。

  各位代表！

  过去一年取得的成绩，是习近平新时代中国特色社会主义思想科学指引的结果，是省委省政府和市委坚强领导的结果，是市人大市政协和社会各界监督支持的结果，是全市广大干部群众奋力拼搏的结果。在这里，我代表市人民政府，向奋战在各条战线上的广大干部群众，向省驻陇南各单位、人民解放军和武警驻陇南部队官兵，向各民主党派、人民团体、工商联、无党派人士、离退休老同志，以及所有关心、支持陇南发展的社会各界人士表示崇高的敬意和诚挚的感谢！

  我们也清醒地看到，我市经济社会发展中还存在一些困难和不足，主要表现在：脱贫攻坚任务艰巨，全市仍有773个深度贫困村、18.43万贫困人口尚未脱贫，分别占全省的20%和17%，全面完成脱贫任务的时间越来越紧、难度越来越大；重大项目储备不足，产业项目偏少，发展后劲不足，促投资、稳增长的压力持续加大；产业转型升级较慢，新兴产业规模较小，优化产业结构、推进高质量发展的任务还很繁重；在教育、医疗、住房、食品药品安全等方面，群众还有一些不满意的地方，改善民生的任务仍然较重；部分干部作风、能力与新时代的发展要求还有差距，不作为、慢作为现象依然突出，等等。解决好这些问题是我们今后工作的目标导向和重点任务，我们一定要正视差距、迎难而上、勇于担当、拼搏实干，为建设幸福美丽新陇南作出更大的贡献！

2019年重点工作

  2019年，是新中国成立70周年，是打赢脱贫攻坚战、全面建成小康社会的关键之年。从政策机遇来看，中央实施了乡村振兴战略、新一轮西部大开发、长江经济带建设等重大战略，财政、货币和金融政策将在扩大内需、调整结构、基础设施领域补短板、服务实体经济等方面发挥更大作用；省委省政府将在打赢脱贫攻坚战、加强基础设施建设、加快中新南向通道建设、推动绿色发展崛起等方面加大工作力度，这些都为我市推进高质量发展提供了良好的机遇和强大的动力。从自身发展来看，通过深化实施“433”发展战略，我市在基础设施建设、特色产业发展、经济转型升级、城乡面貌改善等方面，实现了重大突破，打下了良好基础，正处在发挥优势、释放潜力、大有可为的重要战略机遇期。我们要准确把握发展大势，坚定发展信心，凝聚合力攻坚，努力在新的一年做出更大的成绩。

  今年政府工作的总体要求是：以习近平新时代中国特色社会主义思想为指导，全面贯彻党的十九大和十九届二中、三中全会精神，统筹推进“五位一体”总体布局，协调推进“四个全面”战略布局，深入落实习近平总书记视察甘肃重要讲话和“八个着力”重要指示精神，全面贯彻中央“五个坚持”“六稳”和“巩固、增强、提升、畅通”方针政策，深入落实省委省政府决策部署和市第四次党代会精神，深化实施“433”发展战略，继续打好三大攻坚战，突出抓好十大生态产业，大力推进基础设施建设，发展壮大县域经济，加快推动乡村振兴，统筹推进稳增长、促改革、调结构、惠民生、防风险各项工作，推动经济高质量发展、城乡协调发展、社会和谐发展，增强人民群众获得感、幸福感、安全感，加快建设幸福美丽新陇南，以优异的成绩庆祝新中国成立70周年。

  全市经济社会发展的主要预期目标是：生产总值增长6%以上，固定资产投资增长6%，规上工业增加值增长3.5%，社会消费品零售总额增长8%，一般公共预算收入增长3%，城乡居民人均可支配收入分别增长7%和9.5%，居民消费价格指数、单位生产总值能耗、污染减排完成既定目标。

  今年重点抓好九个方面的工作：

  一、全力冲刺打好脱贫攻坚战

  今年是脱贫攻坚工作非常关键的一年，对打赢脱贫攻坚战具有决定性意义。我们要聚焦精准脱贫目标任务，坚持基础性工作与产业扶贫两手抓，确保武都区、康县、文县达到整县退出验收标准，570个贫困村脱贫退出，稳定脱贫10万人以上，为全面完成脱贫攻坚任务打下决定性基础。

  精准完善脱贫攻坚“一户一策”。把“一户一策”作为精准脱贫的重要抓手，根据贫困群众家庭实际情况，对照脱贫验收标准，按照扶贫产业发展要求，及时更新完善信息，进一步明确贫困户脱贫增收的目标任务和具体措施，细化靠实帮扶责任，真正把“一户一策”变成精准脱贫的任务书、作战图，使贫困户自我发展有信心、干部帮扶有靶心。

  全面补齐基础设施短板。加强扶贫资金精准投入，加大深度贫困村补短板的力度，努力完成今后两年的基础设施建设任务。全面完成交通扶贫兜底性指标，积极硬化一批自然村道路。充分发挥水务投资公司作用，基本完成农村饮水安全巩固提升任务。全面完成剩余3867户危房改造，彻底消除农村危旧房。加快完成9377户易地扶贫搬迁扫尾工程，积极推进旧房拆除、生态恢复、就地就业等后续工作，年底前全部搬迁入住，让搬迁群众既能安居又能乐业。加快实施新一轮农村电网改造升级工程，贫困村基本实现稳定可靠的供电服务全覆盖。整修2万户非贫困户庭院，全面提升贫困村整体形象。

  大力推进扶贫产业革命。产业扶贫是今年脱贫攻坚的重中之重。要拓宽扶贫产业发展思路，投入10亿元信贷资金，调整种养殖结构，做好产业的“加减法”，加快发展高效脱贫产业。科学制定县乡扶贫产业规划，精准制定各村各户扶贫产业发展计划，大力发展每亩3000元以上收益的种植业、每户10000元以上收益的养殖业，在每个贫困村建设产业示范点，在成规模的村建设产业园，在集中连片贫困区域建设产业带，以高收益、规模化的产业支撑贫困户持续稳定脱贫。加强农业基础设施建设，增加农业科技含量，提高科学管理水平，提升农产品附加值。积极发展建筑、旅游、采砂及食品、服装、饲料加工等非农产业，积极扶持小家禽、小庭院、小作坊、小手工、小买卖“五小”产业发展，推动扶贫产业发展多元化。因地制宜发展效益前景好、带贫能力强的“扶贫车间”，建设100个“扶贫车间”示范点。坚持产业扶贫带动发展村级集体经济，通过资产盘活、资源开发、多元合作等方式，确保每个贫困村集体经济收入达到万元以上。

  创新产业发展机制。健全完善龙头企业、合作社和贫困户的利益联结机制，真正把产业带起来、让群众富起来。发挥好富民公司在产业扶贫中的领头雁作用，大力推广“宕昌模式”，带动扶贫产业规模发展。通过自建、外引等方式，培育发展龙头企业，依托富民公司吸引民营企业合资，实现龙头企业对合作社全覆盖。积极推广轻资产合作模式，确保宕昌县、礼县、西和县德青源金鸡产业项目建成投产。加快合作社晋等升级，积极落实政策待遇，鼓励支持事业单位人员、未就业大中专毕业生、企业经营管理人才到贫困村领办创办合作社，对合作社带头人进行全覆盖培训，创建示范合作社500个，提高合作社带动能力。加大电商扶贫力度，扩大精品网店覆盖面，提高电商营销带动力，创建电商扶贫示范店1000个。加大产业资金投入力度，管好用好特色产业发展工程贷款、农产品收购贷款、产业发展投资基金、到户产业扶持资金，充分发挥资金的杠杆作用。进一步扩大政策性农业保险品种和覆盖面，有效降低自然灾害和市场波动风险。

  巩固提高公共服务保障水平。扎实做好教育扶贫，加大控辍保学力度，九年义务教育巩固率达到96.5%，确保贫困家庭学生全部资助、适龄人口全部入学。全面落实健康扶贫政策，推动基本医保、大病保险、医疗救助和签约医生覆盖全部贫困人口，认真落实“先诊疗后付费”和“一站式”结报等政策，全面建好贫困村卫生室。统筹抓好社会保障兜底，不断提高城乡低保水平，推进贫困人口基本养老保险全覆盖，加快建立贫困家庭“三留守”关爱服务体系，推进农村低保制度与扶贫政策有效衔接，确保全面小康路上不漏一户、不落一人。发挥好农村公益性岗位作用，新增城镇公益性岗位500个，促进贫困户未就业大中专毕业生就业。加大精神扶贫力度，完成精准扶贫劳动力培训4万人，增强贫困群众内生动力。

  深化东西扶贫协作。积极推进“青企入陇”“陇货入青”行动，扎实做好青岛企业投资的现有项目建设，争取每个县区引进2户青岛企业、在对口帮扶市区设立一个特色产品专卖店，进一步加大人才培训、劳务输转等方面的协作，大力落实城乡土地增减挂钩指标跨省域交易政策，推动中央定点帮扶单位扶贫项目、资金、措施落地见效，扎实开展“百企帮百村”精准扶贫行动，形成社会全力帮扶的良好局面。

  二、千方百计保持经济稳定增长

  积极应对经济下行压力加大、稳中有变、变中有忧的严峻形势，坚持稳中求进，大力推进产业带动、投资拉动、消费驱动，保持经济平稳健康发展。

  大力发展生态产业。坚持走绿色发展之路，加快实施十大生态产业带动性项目，推动经济高质量发展。大力发展循环农业，加快循环农业示范县、示范点建设，抓好20个田园综合体、蓝莓种植和油橄榄产业融合发展示范园建设，实施粮改饲2万亩，新建果蔬保鲜库42座，推广种养加一体化全产业链生产。发展壮大中医中药产业，统筹推进基地建设、精深加工、品牌打造、多元营销，延长产业链条，提高企业效益，加快国药控股公司半夏产业园、大黄产业园在西和县、礼县落地实施，推广中药材标准化种植50万亩。加快推进旅游强市战略，坚持“大旅游”和“小旅游”齐抓共进，以大视野、新理念做好全域旅游规划，抓好宕昌县、两当县、康县、成县全域旅游示范县建设，加快官鹅沟5A级景区和文县白马河民俗风情旅游4A级景区创建工作，大力推进茶马古镇、盐官古镇、哈达铺红军街、天池景区等旅游重点项目建设，积极完善景区道路、停车场、游客接待中心、标准化旅游厕所等基础设施；加快发展乡村旅游，着力培育绿色采摘、特色餐饮、养生度假等体验项目，创建乡村旅游示范村20个；促进文化与旅游深度融合，办好乞巧女儿节、白马人民俗文化旅游节、宕昌冰雪旅游节等活动，打造一批旅游精品路线和品牌，放大旅游综合效应。大力发展数据信息产业，积极推进大数据基础设施建设，拓宽陇南大数据云计算中心应用领域，发展区块链、质量链等大数据应用产业。加快推进电子商务提档升级，深化“1333”电商发展思路，聚力打好电商发展整体战、融合战、质量战和全域战，加快创建国家电子商务示范城市；加强与阿里、腾讯、京东、苏宁等电商大平台对接合作，培育销售额千万元以上的示范网店100户，建立200支专业化电商团队；做大做强供销电商，推进“供销e家”市县乡村网络全覆盖；大力实施“全企入网”工程，不断拓宽电商应用领域，提高电商应用水平，让陇南电商遍地开花结果。积极培育发展节能环保、清洁生产、清洁能源等产业，大力发展风电、光电、生物质等新能源，建成武都千坝风电一期工程，促进经济转型升级。

  加快基础设施项目建设。认真落实项目投资清单管理制和领导包抓责任制，坚持一事一议解决具体困难和问题，全力推进项目建设，以大投资、大项目带动大发展。盯紧重大项目抓投资，谋划实施投资规模500万元以上项目870个、总投资1100亿元，力争年内完成投资230亿元以上。抢抓国家基础设施领域补短板政策机遇，围绕10项重点任务，谋划储备一批重大项目，争取纳入国家、省上重大项目盘子。完善立体综合交通体系，加快推进洛门至礼县、余家湾至凡昌、望关至宁强等三条地方高速公路前期工作，力争上半年开工建设洛礼高速，年内开工余凡高速，加快推进望关至宁强高速，论证规划成县至天水秦州高速公路；武九高速完成年度投资计划，两徽、渭武高速陇南段建成通车，陇南综合客运枢纽建成投运，争取天水至陇南铁路列入国家西部大开发规划；加快武都区、礼县、宕昌县3个通用机场和4个直升机起降点前期工作，积极拓展成县机场航线，加快推进陇南至昆明等航线开通工作，力争旅客吞吐量达到20万人次；抓好龙凤大桥至西礼大桥等6条省道建设项目，宕昌县好梯至韩院等3条省道改建项目年内建成通车，加快宕昌至官鹅沟、成县西狭颂景区旅游公路建设，全面修复水毁道路，使陇南的交更加四通八达。进一步完善水利基础设施，抓好节水灌溉和江河干支流、中小河流、山洪沟道治理等水利项目建设，开工建设徽县宋家湾水库和两当县徐杨河调水工程，继续实施好“五小水利”工程，切实提升水利保障能力。抓好能源通信项目建设，开工建设陇南段800千伏输电工程，建成徽县330千伏变电工程，新建4G基站110座，实现4G网络全覆盖。

  扩大提升消费市场。大力发展通道物流经济，抢抓“一带一路”和中新南向通道物流节点建设机遇，依托兰渝铁路、陇南经济开发区，加快建设大商贸、大物流体系。加快推进陇南空港物流园、物流集散中心项目建设，开工建设武都花椒市场、西和果蔬批发市场、徽县栗川便民市场、宕昌理川中药材市场等项目，抓好西和海通现代物流园、成县顺通电子商务物流园等冷链物流项目，加快构建现代商贸物流体系。围绕高铁经济带建设，及早规划建设好哈达铺、吉石坝等现代物流园。大力发展信息技术、金融保险、养老服务、人力资源服务等现代服务业，加快推进“互联网+ ”在教育、医疗、旅游等领域融合运用，提高消费领域智能化、网络化、数字化水平。

  加大招商引资力度。积极“走出去、请进来”，全面加强对外合作交流，促进项目落地实施，提升招商引资质量水平。围绕农产品加工、绿色有机食品生产、中医药开发、现代物流、文化旅游、新材料制造、现代服务业等产业，瞄准500强企业、行业领军企业等，大力开展党政领导带头招商、小分队上门招商、重点区域驻点招商等精准招商活动。加强与万达集团、广东恒大、天能重工、龙浩集团等已有合作意向企业的精准对接，继续举办PPP项目和招商引资项目集中推介活动，积极参加兰洽会、西洽会、西交会等重大节会，加大优势资源、优势产业、优势项目宣传推介，认真落实优惠政策，吸引更多的战略投资企业来陇南落户发展，做到签约一个，开工建设一个，力争到位资金增长6%以上，民间资本和社会投资增长10%以上。

  三、集中力量壮大县域经济

  县域经济是富民强县的重要支撑。我们要把县域经济作为加快发展的重要抓手，加大经济总量，提升发展质量，推动优势产业集群发展，使县域经济实力越来越强。

  做大做强工业经济。工业是县域经济的支柱产业，是实体经济的主体。坚持工业强市战略，充分发挥区域优势，围绕主导产业，科学布局县域工业，坚持改造提升传统产业和培育壮大新动能两手抓，推动工业规模、质量、效益跃上新台阶。今年落地实施20个以上重点工业项目，完成投资10亿元，培育入规企业5户以上，规上工业企业实现利润22亿元以上。着力推进工业转型升级，加快有色冶金、建筑建材、水电能源等传统产业向中高端迈进，提升产业链水平，打造绿色工业品牌，力争建成15家绿色矿山。支持企业实施技术装备提升、智能化改造和产品升级换代等项目，力争天顺公司植物提取等8个项目年内建成投产；抓紧完成万利公司25万吨铁合金、红川酒业2000吨白酒技改扩能等项目前期工作；及早论证实施宝徽公司10万吨锌冶炼技改等重大工业项目，不断增强工业发展后劲。抓好重点骨干企业生产要素保障，创建中药材、白酒酿造、橄榄油、银杏等技术开发中心，促进企业稳产高产、提质增效。

  加快培育新的经济增长点。按照全市经济中心和产业中心的定位，全面推进陇南经济开发区建设，努力把陇南经济开发区建成产城融合新区。大力支持开发区道路、污水垃圾处理、供排水等基础设施项目建设，跟踪推进陇南汉能移动能源产业园建设，加快天诚汽车城、物流园、商旅小镇等项目建设进度，开工建设东滨河路、红川大道、金鑫新材料加工、青岛利和生物萃取等项目，确保西滨河路、污水处理工程、科创孵化园等项目建成投用。按照全产业链发展要求，尽快布局开发区产业，加快园区招商引资力度，把陇南经济开发区建成全市产业集群区。积极发展“飞地经济”，引进5户以上大企业、大集团落户开发区，每个县区谋划2个投资亿元以上的工业项目落地开发区。理顺开发区管理体制机制，制定出台融资、土地绿色通道、政府债券等优惠政策，为入园企业提供良好的服务。加快各县区经济开发区、工业园区建设，配套完善基础设施，带动县域经济振兴发展。大力发展沿河经济带，推广康县太石乡统筹推进河道治理、生态文明建设、发展沿河经济的经验做法，培育新的县域经济增长点。

  大力支持民营经济发展。民营企业是发展县域经济的主要力量。深入贯彻落实习近平总书记在民营企业座谈会上的重要讲话精神，不折不扣落实支持非公经济发展19条、中小微企业高质量发展58条等政策措施，认真落实领导干部联系民营企业和商会制度，建立健全全程代理服务机制，实行“保姆式”“贴近式”“零距离”跟踪服务，加快构建亲清新型政商关系。进一步减轻企业税费负担，真正为民营企业排忧解难，营造法治化、便利化营商环境，保护民营企业家人身和财产安全，让民营企业家放心投资、舒心创业、安心发展。新增中小微企业300户以上。鼓励支持符合条件的中小企业通过“新三板”和区域股权市场直接融资，进一步拓宽融资渠道，提高土地、房产、林权、知识产权等抵质押率，扩大信贷投放，下决心解决一批企业融资难的问题。大力支持企业开拓产品市场，加大在北京、上海、广州、青岛等城市的推介宣传，办好茶博会、药博会、特色产品展销会及花椒、核桃、苹果等农特产品展销活动，全力以赴把陇南特色产品打出去。

  四、统筹协调推进城镇建设

  突出生态之城、活力之城、陇蜀之城的城市定位，加快构建以县城为中心，中心城镇、特色小镇协调发展的城镇建设体系，着力打造秀美宜居的城镇家园。

  修编完善城乡规划。坚持城乡一体发展，加快推进城乡功能区、土地利用、环境保护等规划“多规合一”，提高规划的前瞻性和科学性。积极推进陇南市中心城区控制性详规及各县城市总规修编工作，抓紧编制各县城市“双修”规划，做好城市公共服务设施专项规划编制工作。突出“一镇一业”“一村一品”，修编完善乡镇、村庄规划。严格执行规划审批制度，强化规划的约束力，做到“规划一张图、建设一盘棋”。

  抓好市政设施建设。加强武都城区建设，统筹推进汉王、吉石坝、两水片区开发建设，开工建设市区有轨电车旅游示范线、长江大道东延伸段二期、汉王一号环路一期工程等市政基础设施；加强各县城区建设，实施一批供气供热、污水垃圾处理、供排水管网改扩建、水源地保护、公园绿地、停车场等城市配套设施项目，整体提升城市建设水平。继续实施城市“双修”工程，优化空间布局，完善城市功能，强化城市综合治理，加快“智慧城市”建设，提升城市品位，提高城市精细化管理水平。加快完善中心城镇基础设施，开工建设投资60亿元的武都汉王橄榄小镇和安化镇改造升级项目，抓好其他9个小城镇改造提升工程，提高中心城镇的城市化水平。

  强化住房保障。坚持综合施策，加强房地产市场管理，合理引导和控制好市县城区房价，促进楼市健康平稳发展，加快建立多元化住房保障体系，完成棚户区改造3525套，实施房地产开发项目34项，力争完成投资28亿元，更好满足居民多层次住房需求。推进农业人口向城市转移，加快户籍制度改革，落实好农村人口进城落户政策，户籍人口城镇化率达到26.5%。

  五、扎实有效推进乡村振兴战略

  围绕农业强、农村美、农民富的目标，按照“五个振兴”的要求，在两当县、徽县、成县和其他县区的非贫困乡村，大力发展特色产业，改善乡村人居环境，革除乡村生活陋习，加强乡村治理，把乡村建设成为产业兴旺、山清水秀、文明祥和的秀美家园。

  大力推进产业振兴。按照“独一份”“错峰头”“好中优”“特别特”的要求，深化农业供给侧结构性改革，大力推进特色产业规模发展、规模经营，提高农业产业效益。推广运用农业新技术、新模式，完成全膜双垄沟播47万亩，落实好粮食安全省长责任制，保持粮食总产稳定增长；推广新品种标准化种植，持续开展提质增效工作，完成经济林综合管理600万亩，规模发展中药材50万亩，使特色产业面积稳定在1000万亩左右；建设标准化规模养殖场30个，养殖中蜂38万箱、牛羊猪182万头、生态放养鸡580万只，提高特色产业发展规模化水平。积极培育壮大规模经营主体，发展规模家庭农场40家，（下转第三版）(上接第二版)规范提升农民合作社3000个，发展龙头企业42个，推行“龙头企业+合作社+产业基地+农户”“龙头企业+合作社+家庭农场”等经营模式，实现对各区域各种特色产业的覆盖带动，提高特色产业经营规模化水平。抓好“三品一标”、集体商标和区域公用品牌创建工作，整合现有品牌，打造特色名牌，提高品牌竞争力。大力培养农村实用技术人才，鼓励支持外出农民工、退役军人、农村大中专毕业生返乡创业，在每个村培养一批“田秀才”“土专家”和致富能手，带动群众脱贫致富。

  着力建设秀美乡村。加强乡村人居环境综合治理，提高乡村绿化美化亮化水平，建设一批宜居乡镇、美丽农村。加快推进小城镇改造提升工程，实施18个乡镇绿化美化亮化工程，整治乱停乱放、乱修乱建，实施拆围植绿，强化特色风貌改造，完善公共服务设施，使更多的小城镇宜居宜游。加快农村生活垃圾和污水处理、畜禽养殖污染治理，推进农村厕所革命，整理房屋庭院，整修村庄道路，建设村内广场，保护古树古院落，大力植树造林、种花种草，让更多的农村展现出山清水秀、鸟语花香、风景如画的田园风光。

  积极弘扬文明新风。以社会主义核心价值观为引领，积极传承弘扬优秀传统文化，培育邻里和睦、勤俭节约、勤劳淳朴的良好新风尚。积极开展文艺小分队送文化下乡活动，编排一些群众喜闻乐见的文艺节目进行巡回演出；组织群众自编自演，在丰收节等节日期间，开展一些积极向上的文化活动，寓教于乐，丰富农村文化生活。建设新时代文明实践中心，挖掘发扬优秀民俗文化，保护传承非物质文化遗产，加强文明健康新风尚教育，改变红白喜事大操大办、天价彩礼等陈规陋习，评选表彰“文明家庭”“致富能手”等先进典型，焕发乡村文明新气象。

  加强乡村治理。坚持法治、德治、自治相结合，健全乡村治理体系，提高乡村治理水平，使乡村充满活力、安宁祥和。加大农村普法力度，加强法治宣传教育，做好法律援助工作，使农民群众知法用法、遵纪守法。加强农民思想道德教育，弘扬传承传统道德观念，使农民群众自觉用道德规范约束自己。加强村民自治，强化村规民约，推行“3+5”群众工作法、“群众说事”、村务公开等民主管理制度，使乡村治理更加有序有效，使农民群众生活得更幸福。

六、坚持不懈抓好环境保护和安全生产

  牢固树立和践行绿水青山就是金山银山的理念，坚持生态优先、保护优先的原则，围绕构建长江上游生态安全屏障，实行最严格的生态保护制度，坚决打好污染防治攻坚战，坚决守住安全生产“底线”，让陇南的山更青、水更绿、天更蓝。

  加强生态建设。积极实施生态环境修复工程，大力开展国土绿化行动，让陇南大地播撒新绿。深入开展自然保护区“绿盾2019”专项行动，加大湿地公园、森林公园管护，完成自然保护区调整工作。启动生态乡村创建活动，抓好重点公益林建设、天保工程管护、退耕还林还草等工作，人工造林30万亩，建设绿色通道350公里，打造一批生态建设示范工程。

  加大污染防治力度。深化生态环境保护管理体制改革，完善生态环境保护责任清单，坚决打好蓝天碧水净土保卫战。抓好重点企业污染源达标整治，强化扬尘污染、面源污染和柴油货车污染治理，继续推进冬季清洁供暖，加强散煤治理，全面完成10蒸吨及以下燃煤锅炉综合整治，确保空气质量持续稳定向好。严格落实河长制、湖长制，加大污水处理设施建设力度，突出重点流域环境综合治理，全面完成县级以上水源地清理整治，确保主要河流水质稳定达标、城乡居民饮水安全。巩固推进全域无垃圾专项治理，加快实施土壤污染防治项目，确保土壤环境治理有效改善。严格环境执法和问责，抓好中央、省级环保督察后续整改工作。采取差别化环境管控措施，严禁简单化执行环保政策搞“一刀切”。深入开展节约型机关、绿色家庭、绿色学校、绿色社区和绿色出行创建活动，逐步形成绿色生活方式。加快推进环境监管能力建设，加强环境风险防控，提高应对突发环境事件处置能力。

  加强安全生产工作。牢固树立“红线”意识，时刻绷紧安全生产这根弦，完善安全生产责任清单，突出道路交通、企业生产、尾矿库等重点行业安全监管，持续推进重点领域安全隐患排查整改，从源头上预防安全事故发生。加强安全生产应急管理，扎实开展各类应急演练，着力推进物联网、云计算等技术在重点行业领域安全监管的应用，提高安全生产信息化水平。加强对城市高层建筑、大型综合体、隧道桥梁、燃气设施等重要基础设施的安全检测维护，推进城市安全发展。加强自然灾害防治能力建设，提高防灾减灾水平，切实保障人民群众生命财产安全。

  七、深入广泛推进改革开放

  坚持改革开放不停步，深入推进各项改革，持续扩大对外开放，不断激发经济社会发展活力，努力实现更高质量、更有效率、更可持续的发展。

  全面推进农业农村改革。巩固提高农村“三变”改革成果，努力实现“三变”改革所有行政村全覆盖。深化农村土地制度改革，严格落实耕地保护制度，探索承包地“三权”分置有效实现形式，加大农村土地、林地流转力度，发展多种形式的适度规模经营。抓好武都区、康县农村集体产权制度改革试点工作，为全市创造经验。进一步完善农村产权交易平台建设，提高规范化运行水平。

  深化财税金融体制改革。扎实推进财税机制改革，加强税收征管，着力培植新的财源项目，探索建立多元化、可持续的财税增长机制；进一步优化支出结构，压缩一般性支出，过紧日子，过苦日子，提升保运转、保民生、保稳定、促发展能力；强化预算编制执行，全面推行绩效管理，规范政府举债融资机制，用好地方政府专项债券，不断提升现代财政管理水平。深化国资国企改革，建立国资监管、投资运营、实体企业三位一体的国有资本管理体系，加快实现管资产向管资本的有效转变。大力推进金融创新，进一步完善市县两级融资担保体系，积极防控化解金融风险，强化金融服务保障；全方位开放城乡基础设施投融资市场，通过出让国有资源资产和资产证券化等多种方式补充基础设施建设项目资本金，加大向金融机构推荐项目力度，着力破解项目资金难题；采取建立资金池、建养一体化、融资租赁、经营性租赁等模式，切实把“沉睡”的资产用足用活、用出效益；在全市行政村设立农村金融综合服务室，提升农村金融服务水平。

  深入推进政务改革。全面深化“放管服”改革，加快推进政务服务“四办”改革，基本实现政务服务事项进驻大厅全覆盖，网上可办率不低于90%，70%以上的事项实现“一窗”分类受理，企业和群众办事提供材料减少60%以上。持续开展“证照分离”改革和“减证便民”行动，积极推行“互联网+商事登记”模式，新登记市场主体1万户以上。全面推行统一的市场准入负面清单制度，全域实行企业投资项目承诺制、代办服务、模拟审批、容缺受理和建设项目区域化评估评审等优化项目审批服务的措施办法，营造良好的营商环境。深化行政执法体制改革，构建权责统一、权威高效的行政执法体制。加快医药卫生、供销合作社等行业改革，进一步激发社会发展活力。

  着力推进对外开放。主动融入中新南向通道建设、成渝经济圈、长江经济带，切实加强与毗邻地区的务实合作，力争在基础设施互联互通、产业协作、区域协同发展等领域取得实质性进展，使陇南真正成为甘肃向南开放的桥头堡。积极融入“一带一路”，巩固拓展与白俄罗斯格罗德诺市合作成果，力争年内互设经济代办处。加强与西班牙、意大利、土耳其等国家合作发展油橄榄产业，积极申报加入国际油橄榄理事会。积极组织参加敦煌文博会、深圳文博会等重大文化交流活动，深化全方位交流与合作，在更大范围展现陇南开放发展的新魅力。

  八、全心全力保障和改善民生

  坚持以人民为中心的发展思想，以满足人民日益增长的美好生活需要为目标，加快补齐民生事业短板，加强和创新社会治理，使发展成果更多、更好、更公平地惠及全市人民。

  统筹发展社会事业。加快城乡教育一体化发展，认真实施全面改薄项目，加快标准化学校建设，办好必要的村学和教学点，提高乡村教育发展水平；优化配置城区义务教育优质资源，建设一批城区优质中小学分校，提升50所乡村中小学教育质量，引进一批优秀教师，进一步解决城区学校“入学难”和“大班额”问题；礼县、康县通过义务教育均衡发展评估；实施好第三期学前教育行动计划，加快普及高中阶段教育，大力发展现代职业教育；深化教育综合改革，大力培养优秀教师，激发教师队伍活力，进一步提高教育教学质量。加快城乡医疗一体化进程，加强大医院和基层卫生院医联体建设，实现城乡医疗资源共享，大力提升50所乡镇卫生院医疗服务水平，方便农村群众看病；加强骨干医师人才培养，引进急需紧缺专业医疗人才，提升医疗健康服务水平，陇南卫校升级取得实质性进展。加快推进养老服务业发展，开展养老院服务质量专项行动，引导社会资本投资养老事业。不断提升公共文化服务水平，创作一批特色鲜明、具有一定影响力的文艺精品；建设“历史再现”工程博物馆6个，建好市区新华书店，市图书馆、市博物馆向市民免费开放。广泛开展全民健身活动，新建村级农民体育健身工程100个。不断完善科技创新和服务平台，大力培育科技服务机构，加大科技创新力度，加快科技成果转化，提高科技贡献率。

  抓好就业和社会保障工作。深入实施“创业推动工程”，应届高校毕业生就业率达到85%以上，城镇新增就业1.75万人，城镇登记失业率控制在4%以内。落实最低工资标准和工资集体协商制度，保障农民工工资支付，完善以社会保险、社会救助、社会福利制度为主体的综合保障体系，提升职工和居民医疗保障服务水平，提高城镇退休人员养老保险待遇和城乡居民基础养老金最低标准，确保城乡居民收入稳定增长。

  加强和创新社会治理。深入推进法治陇南建设，全面开展“七五”普法工作，创新农村社区治理机制，发挥好村民自治作用，深化信访制度改革和信访法制化建设，健全完善矛盾纠纷多元化解机制。持续开展食品药品安全专项整治，保障人民群众饮食用药安全。做好双拥共建和优抚安置工作，增进军政军民团结。加强民族宗教事务管理，深入广泛开展民族团结宣传教育和创建工作，推进老龄、残疾人、档案和公益事业发展，大力支持各民主党派和工、青、妇等群团工作，积极做好留守老人、留守儿童和妇女儿童权益维护工作。深化“平安陇南”建设，深入推进“扫黑除恶”专项斗争，依法打击和惩治各类违法犯罪活动，切实维护社会大局和谐稳定。

  大力兴办民生实事。群众利益无小事，一枝一叶总关情。坚持把人民群众的小事当作政府的大事，从人民群众关心的事情做起，从让人民群众满意的事情做起，尽最大努力为群众办实办好文化惠民工程、便民惠民暖心工程、居民使用天然气补贴、困难弱势群体法律援助、农村留守儿童意外伤害险、农村危房改造、农村饮水安全、县乡农贸市场建设等8件民生实事，让人民群众的生活越来越幸福。

  九、从严从实加强政府自身建设

  做好政府工作，加强自身建设是保障。我们要聚焦忠诚干净担当、为民务实清廉，坚持“实”字当头、“干”字为先，切实提升政府工作效能和水平，努力建设人民满意的政府。

  树牢“四个意识”。深入学习贯彻习近平新时代中国特色社会主义思想，牢固树立“四个意识”，坚决做到“两个维护”，严守政治纪律和政治规矩，在政治立场、政治方向、政治原则、政治道路上同以习近平同志为核心的党中央保持高度一致。坚持以党的事业、人民的利益和陇南的发展大局为重，坚决贯彻落实中央，省委、省政府和市委的各项决策部署，使政府工作再上新台阶。

  推进依法行政。严格遵守宪法法律，坚持把法治思维和法治方式贯穿于政府工作全过程，加快建设法治政府。进一步完善政府议事决策规则，坚持政府法律顾问和民主决策制度，严格按法定权限和程序开展工作，提高政府工作科学化、民主化、法治化水平。自觉接受市人大的法律监督和市政协的民主监督，广泛听取各方面的意见建议，提高人大代表建议和政协提案办理质量，让政府工作更好体现民意。

  勇于担当负责。坚决贯彻省委、省政府和市委转变作风有关规定，积极践行“三严三实”，持之以恒纠正“四风”，坚决整治不担当、不作为、懒散慢等作风问题，着力营造干事创业、担当作为的浓厚氛围，推动政风行风持续好转。健全完善协调顺畅的工作运行机制，建立任务清单和责任清单，严格落实定事定责限期办理制度和责任分解机制，加大政务督查督办力度，确保件件有着落、事事有回音。大力发扬雷厉风行、敢于碰硬、真抓实干的好作风，真正干出实打实的新业绩。

  坚持廉洁从政。严格落实党风廉政建设主体责任，严格执行中央八项规定实施细则，加强政府系统廉政风险防控，发挥好市政府重大行政决策廉政监督委员会作用，强化审计监督，加大对精准扶贫、重大项目、政府采购、招投标、土地管理等方面的监督管理，严肃查处发生在群众身边的不正之风和腐败问题，坚持严于律己、廉洁修身，干干净净干事，努力建设清正廉洁、人民满意的好政府。

  各位代表，新起点孕育新希望，新征程要有新作为。我们要更加紧密地团结在以习近平同志为核心的党中央周围，在省委、省政府和市委正确领导下，凝心聚力，开拓进取，攻坚克难，努力完成今年经济社会发展目标任务，为全面建成小康社会、建设幸福美丽新陇南而努力奋斗！
